# Trajectories of depressive symptoms in young and middle-aged men who have sex with men with new HIV-diagnosis: a 1-year prospective cohort study in Beijing, China

**DOI:** 10.3389/fpubh.2023.1244624

**Published:** 2023-10-17

**Authors:** Xiao Li, Yu Liu, Jing Han, Keke Lin, Xiaoyan Bai, Fengling Lu

**Affiliations:** ^1^School of Nursing, Beijing University of Chinese Medicine, Beijing, China; ^2^State Key Laboratory of Experimental Hematology, National Clinical Research Center for Blood Diseases, Haihe Laboratory of Cell Ecosystem, Institute of Hematology & Blood Diseases Hospital, Chinese Academy of Medical Sciences & Peking Union Medical College, Tianjin, China; ^3^Beijing Ditan Hospital, Capital Medical University, Beijing, China; ^4^School of Medicine, Qingdao Huanghai University, Qingdao, China

**Keywords:** HIV infection, men who have sex with men, young adult, middle-aged adult, depression, latent class growth analysis

## Abstract

**Introduction:**

Due to the sexual orientation and HIV diagnosis, young and middle-aged men who have sex with men (MSM) with new HIV-diagnosis may experience more depressive syndromes and face greater psychological stress. The study explored trajectories of depressive symptoms of young and middle-aged MSM within 1 year after new HIV-diagnosis and analyze the related factors.

**Methods:**

From January 2021 to March 2021, 372 young and middle-aged MSM who were newly diagnosed as HIV-infection were recruited in two hospitals in Beijing. Self-rating Depression Scale was used to measure the participants’ depressive symptom in 1st month, 3rd month, 6th month, 9th month and 12th month after HIV diagnosis. The latent class growth model was used to identify trajectories of the participants’ depressive symptoms. Multinomial logistic regression was used to analyse factors related with the trajectories.

**Results:**

Three hundred and twenty-eight young and middle-aged MSM with new HIV-diagnosis completed the research. Depressive symptom in 328 young and middle-aged MSM was divided into three latent categories: non-depression group (56.4%), chronic-mild depression group (28.1%), and persistent moderate–severe depression group (15.5%). The participants assessed as non-depression (non-depression group) or mild depression (chronic-mild depression group) at the baseline were in a non-depression state or had a downward trend within one-year, and the participants assessed as moderate and severe depression (persistent moderate–severe depression group) at the time of diagnosis were in a depression state continuously within 1-year. Multinomial logistic regression analysis showed that, compared with the non-depression group, monthly income of 5,000 ~ 10,000 RMB (equal to 690 ~ 1,380 USD) was the risk factor for the chronic-mild depression group, and self-rating status being fair/good and self-disclosure of HIV infection were protective factors for the persistent moderate–severe depression group while HIV-related symptoms was the risk factor.

**Conclusion:**

Depressive symptoms in young and middle-aged MSM is divided into three latent categories. Extra care must be given to young and middle-aged MSM assessed as moderate or severe depression at the time of HIV-diagnosis, especially to those who had poor self-rating health status, did not tell others about their HIV-infection and experienced HIV-related symptoms.

## 1. Introduction

The MSM (men who have sex with men) population refers to men who have sex with men, gay men, and some bisexual men who exhibit homosexual behavior (1). MSM are a high-risk group for HIV infection due to their multiple sexual partners and low condom use (2). The proportion of newly diagnosed HIV-positive people whose disease was transmitted by gay behaviors increased from 9.1% in 2007 to 23.3% in 2019 (3). HIV infection is a stressful and traumatic experience which has a devastating impact on the mental health of patients, especially in MSM who have already been under some pressure due to their sexual orientation. HIV-positive MSM are a vulnerable social group susceptible to prejudice and discrimination (4). They are more prone to suffer mental health problems, including depression, than the general or other HIV-positive populations. Studies have shown that the incidence of depression in HIV-positive MSM ranged from 21.2 to 92.5% (5–7). Depression led to adverse health outcomes, such as accelerated disease progression, decreased quality of life, and increased mortality (8, 9).

For HIV-positive patients, ages between 18 and 50 are usually considered as young and middle ages (10). The majority of MSM are at this age range when they are diagnosed with HIV (11). Young and middle-aged people are the main labor force in society and are also in the active period of marriageable age and sexual life. However, for MSM, sexual orientation has affected their marriage status to some extent (12). Since traditional Chinese culture regards unmarried or childless people as losers and unfilial (especially in the marriageable age of young and middle-aged people), young and middle-aged MSM face greater psychological pressure, and the incidence of depression may be higher (4, 13). When HIV infection is confirmed, the depression will be further aggravated (14). Thus, the young and middle-aged MSM with new HIV-diagnosis deserve further study.

Increasing experience and gradual changes in life may affect the degree of emotional despondency of young and middle-aged MSM, causing depression to vary at different stages after HIV-diagnosis. It was reported that the status of depression in MSM changed over time, especially in the first year after HIV diagnosis (15). Therefore, measuring their depression once cannot reflect the overall state of depression in any given period. However, most studies on depressive symptom among MSM with HIV-infection are cross-sectional (16–18). In the limited longitudinal studies conducted on trends of depressive symptom of MSM with HIV-infection, findings are mixed. The level of depression was increased in 24 months (19), declined (15, 20) or increased first and then decreased in 12 months (21). The MSM included in these studies experienced different course of HIV-infection. None of them targeted young and middle-aged MSM newly diagnosed with HIV-infection. The discrepancy in these studies may be caused by differences in sample characteristics, particularly the different stages of HIV-infection. Moreover, these longitudinal studies reported group averages of depression, without considering the possible effects of individual differences.

Bonanno, a clinical psychologist, pointed out that individuals show different trajectories of change in their psychological state after a traumatic experience (22). For HIV-positive men, Kelso-Chichetto et al. (23) used group-based trajectory models to identify a four-group depressive symptoms pattern for them (low: 34%; moderate: 34%; high: 22%; severe: 10%). Bengtson et al. (24) also found four depressive symptoms trajectories for HIV-Infected men (low: 61%; mild/moderate: 14%; rebounding: 5%; improving: 13%; severe: 7%). But none of these studies take the MSM into account. Given the special nature of newly diagnosed HIV-positive young and middle-aged MSM, it is necessary to identify trajectories of depressive symptoms over time for them and to define characteristics of individuals within distinct trajectories, so as to adopt targeted interventions. Thus, this study aimed to identify distinct trajectories of depressive symptoms and explore clinical profiles related to the classifications among young and middle-aged MSM within the first year after HIV-diagnosis.

## 2. Materials and methods

### 2.1. Sample

This was an observational cohort study. Young and middle-aged MSM with new HIV-diagnosis were recruited at two specialized-hospitals of infectious diseases in Beijing from 1st January 2021 to 31st March 2021, and then followed them up to 12 months. HIV-positive patients are recommended to return to the hospital at 3-month intervals for follow-up in China, so participants who enrolled in this cohort study were investigated in the first month (no more than 30 days), 3rd month, 6th month, 9th month, and 12th month after being HIV-diagnosed. The last follow-up was conducted on March 31, 2022.

**Figure 1:**
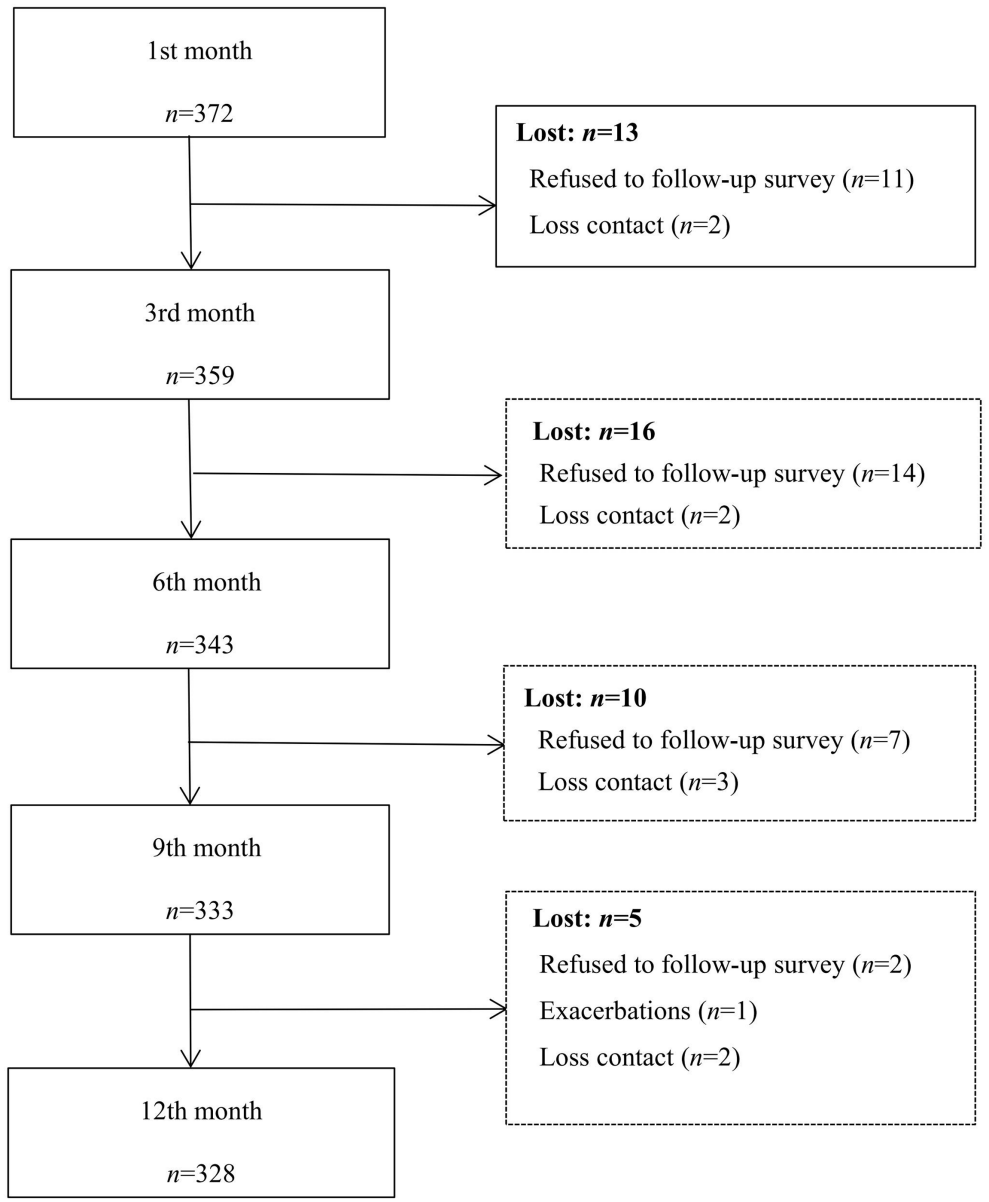
Participation of young and middle-aged MSM in five times survey.

Participants were MSM diagnosed as HIV-infection within the past 30 days, 18 ~ 50 years old, MSM transmission (self-reported). Those who had other serious diseases or had taken psychoactive medication during the past month were excluded. Criteria for discontinuing follow-up were: (1) unexpected event occurred and could not continue to participate in the study; (2) times of not participating in follow-up survey >1. Questionnaires with more than 10% of missing items, or a logical error, or completion time less than 10 min were regarded as invalid. Finally, 328 participants completed the entire process. Figure 1 showed the participants enrollment process. This study was approved by a specific Medicine Ethics Committee (blinded for peer review).

### 2.2. Measurements

#### 2.2.1. Self-rating depression scale

Depressive symptoms were measured by the Chinese version of Self-rating Depression Scale (SDS) (25, 26), a self-assessment scale to measure the degree of depression, which consists of 20 items using a 4-point Likert scale according to the frequency of depressive symptom over a 2 week period, from ‘1’(no or little time) to ‘4’(most or all of the time), and 10 items are reverse scored. The total score ranges from 20 to 80, with a higher score indicating more severe depressive symptom. For Chinese populations, a cut-off standard score of 53 (total scale score by 1.25 is the China’s standard score) has been recommended to screen for depression, and depression is divided into three levels: 53–62 is mild depression, 62–72 is moderate depression, ≥72 is severe depression (27). Cronbach’s α coefficient and split-half reliability of the SDS are more than 0.8 (28). The Cronbach’α coefficient was 0.858 in this study. SDS was collected at both baseline and four times follow-up.

#### 2.2.2. Socio-demographic information sheet

The socio-demographic information sheet was used to collect the participants’ socio-demographic characteristics in the first month after HIV-diagnosis. It includes marital status, occupation status, educational background, living alone or not, have children or not, household location (urban/rural), monthly income (RMB), medical payment, self-rating health status, sexual orientation (homosexual, heterosexual, bisexual), and self-disclosure of HIV infection or not.

#### 2.2.3. Clinical information sheet

Clinical characteristics were collected from the medical record in the first month after HIV-diagnosis. These included CD_4_^+^ counts (an important indicator to observe the efficacy of anti-AIDS treatment), HIV-related diseases (syphilis, condyloma acuminate, tuberculosis etc.), and HIV-related symptoms (continuous fever for over one-month, continuous cough for over one-month, etc.).

### 2.3. Data collection

The first survey was conducted face-to-face for all the participants. Researchers explained the purpose and content of the study in a case-management room in the hospital. After giving the consent, the participants filled in questionnaire. When the patients had questions about the questionnaire, the researchers provided appropriate explanations. The questionnaire was retrieved on the spot after completing it. Due to the pandemic of COVID-19, 193 participants did not visit hospitals for next four times follow-up. Therefore, they completed the survey either by phone or online (Wenjuanxing - a platform providing functions equivalent to Amazon Mechanical Turk). Participants received a gift (10 RMB, equal to 1.4 USD) in appreciation for their participation after each questionnaire.

### 2.4. Statistical analyses

Data analysis was conducted by SPSS 26.0, SAS 9.4 and Mplus 7.0. Statistical analysis of socio-demographics and clinical data was performed using descriptive statistical methods (median, number, and percentage). For comparison of the baseline characteristics of participants who completed the five-time surveys and those who dropped out, non-continuous variables were analyzed using Chi-square tests, whereas continuous variables were analyzed using Mann–Whitney tests.

The latent class growth model (LCGM) assumes that growth parameters are homogeneous within a latent subgroup, making it an extension of growth mixture models (GMM), so the data were analyzed using GMM and LCGM in our study. The model setting begins with a baseline model (single-category growth model), and then gradually increases the number of categories of the model. The best fitting model was chosen by comparing the fit indices among the different categories of models, the realistic meaning or interpretability, and relevant theories. Fit indices used to evaluate the model included: (1) information evaluation index, Higher values for Akaike Information Criterion (AIC), bayesian information criterion (BIC), and sample size-adjusted BIC (aBIC), all of which indicate better fit into the data for the model; (2) the Lo–Mendell–Rubin likelihood ratio test (LMR) and Bootstrapped likelihood ratio test (BLRT), which show a superior model for given k latent subgroups when comparing model-data fit to a model with k-1 latent subgroup; and (3) entropy, which is used to evaluate the classification accuracy of the model. The entropy coefficient has a value between 0 and 1, and the closer the value to 1, the more accurate the classification is. The entropy was calculated with values of ≥0.80, indicating more than 90% were assigned the correct classification (29). The fit indices and substantial meaning were used to determine the final model classification (30). Finally, the effect of participants’ socio-demographic and clinical data on the different track of depression was examined by using multiple classification logistic regression analysis.

## 3. Results

### 3.1. Sample characteristics

A total of 423 young and middle-aged MSM with new HIV-diagnosis were recruited, 51 refused to participate in the survey for personal reason and 372 completed baseline survey. Finally, 328 (88.17%) completed four follow-up surveys (Figure 1).

There were no significant differences in most baseline factors between 328 patients who completed the follow-up survey and 44 patients who did not, except for marital status, have child or not, and have HIV-related diseases. Participants lost to follow-up were more likely to be unmarried, without children and with HIV-related diseases than those who completed the study.

All the 328 participants who completed five follow-up surveys were on average 30.5 years old (19 ~ 50 years old), the majority were single (80.8%), lived alone (41.5%), had no children (89.6%), were employed (70.4%), had education background of college or higher (75.3%), and more than half (70.4%) reported a monthly income of 5,000 RMB (equal to about 690 USD) or more. Hundred and ninety-three patients registered permanent residence as urban. 42.7% used self-paid medical treatment, and more than half (54.9%) did not disclose their infection status to others within a month of HIV diagnosis. Only 119 participants (36.3%) reported good self-rating health status, and 229 participants (69.8%) reported same-sex sexual orientation. Regarding disease-related characteristics, the average of CD_4_^+^ cell counts was 333.35 cell/mm^3^, 245 patients (74.7%) had no other associated disease and 49.7% of them had HIV-related symptoms.

### 3.2. Trajectories of depressive symptoms

Taking the SDS scores of participants at five time points as the observation index, 328 patients were included in the model statistics. First, LCGM was used to fit the data, and the model was configured as a time-parameter free estimate model, with categories 1 to 5 extracted successively. When the number of extracted categories was increased from 1 to 3, the *p*-values of LMR and BLRT were significant (*p* < 0.05), the values of AIC, BIC, and aBIC became smaller, and the entropy value was always greater than 0.8, which led to the conclusion that the retention of three categories was supported by the analysis results. When the number of categories was increased further, the entropy value remained greater than 0.8, the *p-*value of BLRT remained significant, the value of BIC continued to decrease, and no minimum value was observed. As a result, the steep slope graph test was used to sort the value of BIC from small to large, and there was a significant inflection point when the category was 3, and the LMR *p* = 0.079 when taking four categories, implying that three categories should be chosen at this time. The *p-*value of LMR was 0.029 when the number of categories was five, but the variability between the conditional category probabilities was greater, with a minimum value of 0.009, corresponding to a *n* (case) of 3; however, the sample’s representativeness may be limited.

**Figure 2:**
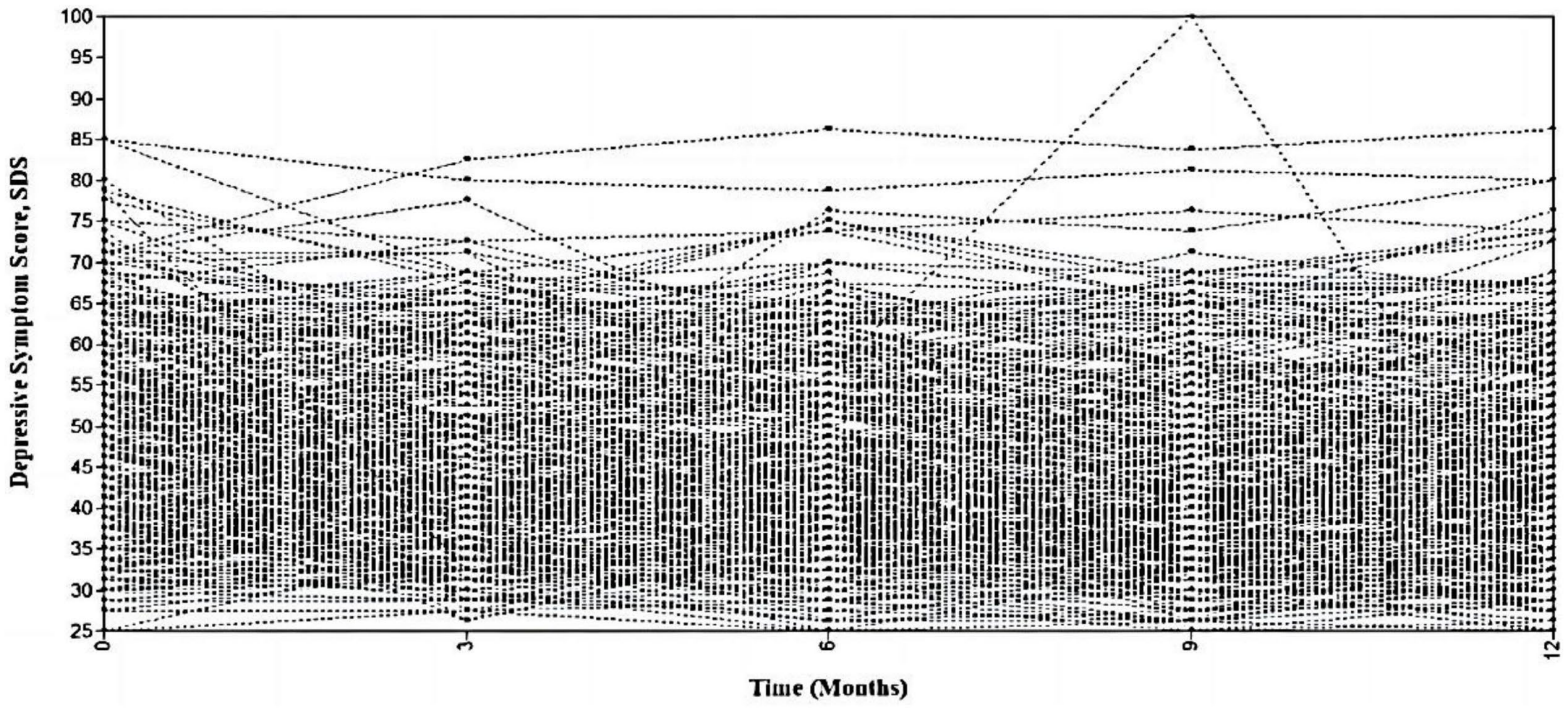
The SDS scores for trajectory observation from all individuals.

The model was set to GMM and re-fitted to determine an even better fitting model. When three categories were retrieved, the BIC value was the smallest, entropy was greater than 0.80, and the *p-*values of both LMR and BLRT reached a significant level, which indicated that three categories remained. Comparing the three categories preserved by LCGM and GMM in further detail, the GMM classification accuracy index entropy value was lower than that of LCGM, and the conditional probabilities of the three categories retained by LCGM were more plausible. Considering the aforementioned indicators and the interpretability of the data, the three best LCGM categories were selected, with 185 cases in Group 1, 92 instances in Group 2, and 51 cases in Group 3, representing 56.4, 28.1, and 15.5% of the total cases, respectively. Table 1 compares the outcomes of LCGM and GMM model fitting evaluations, and Figure 2 depicts the growth trend.

**Table 1:** Comparison of model fit evaluation results for different categories.

Model		*K*	AIC	BIC	aBIC	entropy	*P*		Class probability
							LMR	BLRT	
LCGM	1C	7	12846.21	12872.77	12850.56	—	—	—	1
	2C	10	11979.76	12017.69	11985.98	0.933	<0.001	<0.001	0.345/0.655
	3C	13	11785.31	11834.62	11793.38	0.879	0.025	<0.001	0.281/ 0.564/0.155
	4C	16	11685.27	11745.96	11695.21	0.839	0.079	<0.001	0.137/0.378/0.225/0.259
	5C	19	11630.77	11702.84	11642.57	0.866	0.029	<0.001	0.137/0.009/0.381/0.216/0.256
GMM	1C	10	11657.11	11695.04	11663.32	—	—	—	1
	2C	13	11619.47	11668.78	11627.55	0.767	0.004	<0.001	0.274/0.726
	3C	16	11598.76	11659.45	11608.69	0.833	0.049	<0.001	0.720/0.244/0.0365
	4C	19	11594.94	11667.01	11606.74	0.871	0.025	0.102	0.280/0.677/0.006/0.037
	5C	22	11592.75	11676.20	11606.41	0.809	0.283	0.308	0.604/0.006/0.040/0.149/0.201

**Figure 3:**
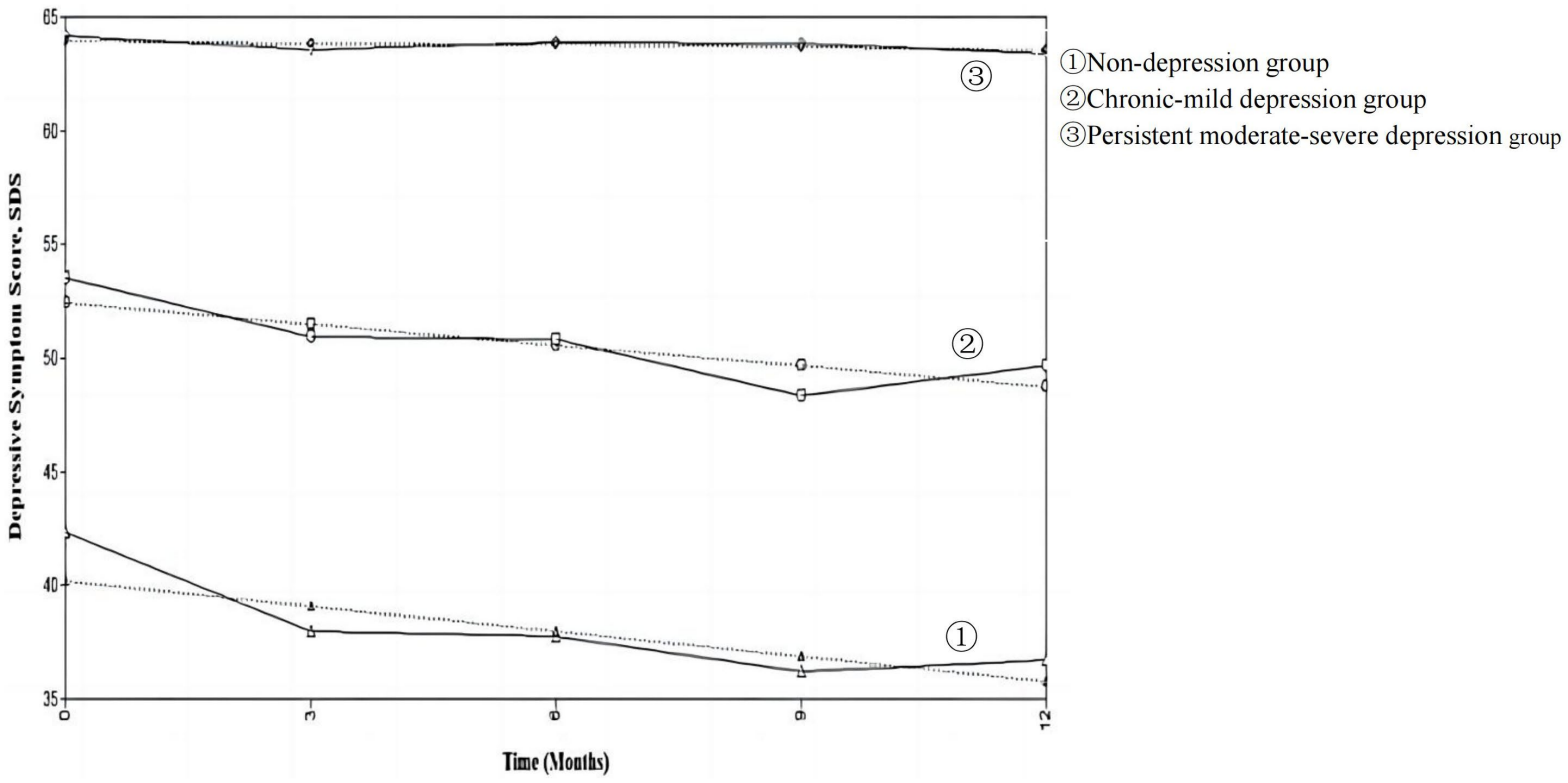
Predicted and observed mean SDS for each trajectory group. Solid lines are mean SDS on rate data, dashed lines are predicted mean SDS based on trajectory modeling.

The designation was based on the trajectory and features of each observation in Figure 2. Figure 3 shows the predicted and observed mean SDS for each trajectory group. Group 1 was no depression in 1 month after diagnosis (*I* = 40.166, *p* = 0.001) and exhibited a declining trend at the following four time-points (*S* = –1.096, *p* = 0.001), so it was named non-depression group. Group 2 was mild depression 1 month after diagnosis (*I* = 52,402, *p* = 0.001) and showed a decreasing tendency at the following four time-points (*S* = –0.917, *p* = 0.015), so it was named chronic-mild depression group. Group 3 had moderate-to-high depression levels in 1 month after diagnosis (*I* = 63.968, *p* = 0.001) and remained stable at the following four time-points (*S* = −0.100, *p* = 0.825), so it was named persistent moderate–severe depression group.

### 3.3. Factors of potential categories of depressive trajectories

According to the results of univariate analysis, the three trajectory categories of depression in the young and middle-aged MSM with new HIV-diagnosis were statistically different in monthly income, self-rating health status, self-disclosure of HIV infection, and HIV-related symptoms, respectively (Table 2 and Table 3).

**Table 2:** Results of univariate analysis of categories of depressive trajectories in young and middle-aged MSM with new HIV-diagnosis (socio-economic variables).

Variable	Non-depression group *N* = 185	Chronic-mild depression group *N* = 92	Persistent moderate–severe depression group *N* = 51	*χ* ^2^	*P*
	*n*(%)	*n*(%)	*n*(%)		
Marital status					
Single	151(81.6)	73(79.3)	41(80.4)	1.248	0.870
Married	22(11.9)	13(14.1)	5(9.8)		
Divorced/widowed	12(6.5)	6(6.5)	5(9.8)		
Having children					
Yes	24(13.0)	8(8.7)	2(3.9)	3.909	0.142
No	161(87.0)	84(91.3)	49(96.1)		
Occupation status					
Employment	132(71.4)	66(71.7)	33(64.7)	6.151	0.188
Unemployment	26(14.1)	14(15.2)	14(27.5)		
Student	27(14.6)	12(13.0)	4(7.8)		
Educational background					
High school or below	39(21.1)	30(32.6)	12(23.5)	4.435	0.109
College or higher	146(78.9)	62(67.4)	39(76.5)		
Living alone					
Yes	71(38.4)	40(43.5)	25(49.0)	2.079	0.354
No	114(61.6)	52(56.5)	26(51.0)		
Household location					
Urban	107(57.8)	55(59.8)	31(60.8)	0.190	0.909
Rural	78(42.2)	37(40.2)	20(39.2)		
Monthly income (RMB / USD)					
<￥5,000 ($690)	56(30.3)	22(23.9)	19(37.3)	13.725	0.008
￥5,000 ~ 10,000 ($690 ~ 1,380)	68(36.8)	53(57.6)	21(41.2)		
>￥10,000 ($1,380)	61(33.0)	17(18.5)	11(21.6)		
Medical payment method					
Medicare-paid	105(56.8)	53(57.6)	20(39.2)	6.530	0.163
Self-paid	76(41.1)	36(39.1)	28(54.9)		
Other	4(2.2)	3(3.3)	3(5.9)		
Self-rating health status					
Poor	12(6.5)	8(8.7)	23(45.1)	69.257	<0.001
Fair	85(45.9)	58(63.0)	23(45.1)		
Good	88(47.6)	26(28.3)	5(9.8)		
Sexual orientation					
Heterosexual	15(8.1)	9(9.8)	5(9.8)	2.220	0.696
Homosexual	134(72.4)	59(64.1)	36(70.6)		
Bisexual	36(19.5)	24(26.1)	10(19.6)		
Self-disclosure of HIV infection					
Yes	94(50.8)	39(42.4)	15(29.4)	7.778	0.021
No	91(49.2)	53(57.6)	36(70.6)		

**Table 3:** Results of univariate analysis of categories of depressive trajectories in young and middle-aged MSM with new HIV-diagnosis (clinical information).

Variable	Non-depression group *N* = 185	Chronic-mild depression group *N* = 92	Persistent moderate–severe depression group *N* = 51	*χ* ^2^ */F*	*P*
	*n*(%)	*n*(%)	*n*(%)		
HIV-related diseases					
Yes	40(21.6)	27(29.3)	16(31.4)	3.117	0.211
No	145(78.4)	65(70.7)	35(68.6)		
HIV-related symptoms					
Yes	74(40.0)	49(53.3)	40(78.4)	24.270	<0.001
No	111(60.0)	43(46.7)	11(21.6)		
CD_4_^+^ counts (cells/mm^3^)	346.00 ± 21.19	335.39 ± 14.26	303.11 ± 188.75	1.319	0.517

Multicategorical logistic regression analysis was conducted with the statistically significant variables in the univariate analysis as independent variables, the potential category of depression as the dependent variable, and the non-depression group as the reference category. Logistic regression analysis showed that, compared with the non-depression group, monthly income of 5,000 ~ 10,000 RMB (equal to 690 ~ 1,380 USD) was the risk factor for the chronic-mild depression group. Self-rating fair/good health and self-disclosure of HIV infection were protective factors for the persistent moderate–severe depression group, and HIV-related symptoms was the risk factor for this group (Table 4).

**Table 4:** Unordered logistic regression analysis of categories of depression trajectories in young and middle-aged MSM with new HIV-diagnosis.

Factor	*β*	*SE*	*Wald χ* ^2^	*P*	*OR*	95% *CI*
**Chronic-mild depression group versus non-depression group**						
Constant	−0.635	0.557	1.299	−0.254	—	—
Monthly income (RMB/USD)						
<￥5,000 ($690)	Ref	Ref	Ref	Ref	—	—
￥5,000 ~ 10,000 ($690 ~ 1,380)	0.632	0.321	3.877	0.049	1.882	1.003 ~ 3.532
>￥10,000 ($1,380)	−0.438	0.382	1.312	0.252	0.646	0.305 ~ 1.365
**Persistent moderate–severe depression group versus non-depression group**						
Constant	0.388	0.572	0.462	0.497	—	—
Self-rating health status						
Poor	Ref	Ref	Ref	Ref	—	—
Fair	−1.631	0.457	12.725	<0.001	0.196	0.080 ~ 0.480
Good	−3.119	0.617	25.526	<0.001	0.044	0.013 ~ 0.148
Self-disclosure of HIV infection						
No	Ref	Ref	Ref	Ref	—	—
Yes	−1.116	0.388	8.248	0.004	0.328	0.153 ~ 0.702
HIV-related symptoms						
No	Ref	Ref	Ref	Ref	—	—
Yes	1.124	0.414	7.375	0.007	3.078	1.367 ~ 6.929

Ref, reference group.

## 4. Discussion

Depression is the most prevalent negative emotion in HIV-positive MSM (31), especially in the first year after HIV diagnosis (15). This study fully considered the heterogeneity of negative emotion groups in newly diagnosed HIV positive MSM, and used LCGM to describe the development process of depression in HIV-positive young and middle-aged MSM, and to describe the growth track of potential categories of depression and from the diagnosis stage to 1 year after diagnosis. This is the first study to describe depressive symptom trajectories among young and middle-aged MSM with new HIV-diagnosis in China. We found that within 1 year after the diagnosis, the depression of young and middle-aged MSM with newly diagnosed HIV-positive had group heterogeneity, and their depression could be divided into three potential groups: non-depression group, chronic-mild depression group and persistent moderate–severe depression group.

This study found that the track of depressive symptoms of young and middle-aged MSM within one-year after HIV-diagnosis is different in terms of severity and stability, which is consistent with previous studies for people living with HIV (23, 32). The classification result of this study is consistent with Bonanno’s (22) view that psychological changes in groups after experiencing trauma follow different trajectories. The non-depression group, the chronic-mild depression group, and the persistent moderate–severe depression group in our study are consistent with the recovery trajectory, the resilience trajectory, and the chronic dysfunction trajectory in Bonanno’s view, respectively. Among the three latent category groups, more than half of the patients (56.4%) were in the non-depression group, which is similar to the results from a longitudinal study on previous HIV-positive patients (32, 33), but higher than the study of Heckman et al. (34) in 105 older adults with HIV infection (31% non-depression) and Larsen et al. (35) in 824 HIV-positive pregnant women (38.5% with persistent no/mild depressive symptoms). The young and middle-aged MSM included in this study were mostly employed (70.43%), single or without children in marital status (89.63%), with high monthly income [70.43% over 5,000 RMB/month (equal to 690 USD)]. With the implementation of the “Four frees and one care” policy in China from 2003, anti-viral drugs, counseling and testing are provided free of charge to more and more patients with AIDS in China, and AIDS patients with difficulties in life are included in the scope of government assistance. This policy is one of the most powerful policy measures for AIDS prevention and control in China. It may to some extent alleviate the stress of AIDS patients, thereby alleviating depression.

Among 328 young and middle-aged MSM with new HIV-diagnosis, 28.1% were in the chronic-mild depression group, which indicated that about one-fourth of patients suffered from mild depressive symptom within 1-month after HIV-diagnosis. However, with the deepening of understanding HIV-infection and experiencing treatment effects, the MSM’s depression symptom decreased and finally became non-depression, which is similar to the findings of Mylona et al. (36) in a study on breast cancer patients within 1 year of diagnosis, suggesting that the young and middle-aged MSM with mild-depression at the time of HIV-positive diagnosis may be able to self-regulate while confronting the stressful event. The challenge model (a type of psychological resilience model) put forward by Garmezy et al. (37) suggests that when an individual encounters adversity of moderate intensity, it will stimulate the potential and adaptability of the individual to cope with stressful events and will further increase its adaptability after the individual has successfully responded.

In this study, 15.5% of new HIV-diagnosis young and middle-aged MSM were in the persistent moderate–severe depression group, which is similar to the “persistently high depression” subgroup (12.7%) of HIV-positive patients using LCGM by Gunzler et al. (33). This finding implied that within a year of receiving an HIV-diagnosis, a small percentage of young and middle-aged MSM experience chronically elevated depression. HIV-positive MSM with depression had lower levels of antiviral drug adherence (38), immunity (39), survival and quality of life (40, 41) compared to those without depression. Depression also increased the prevalence of high-risk sexual behavior (42). It is suggested that medical staff should give psychological care to minimize depressive symptom in young and middle-aged MSM with moderate–severe depression at onset of HIV diagnosis.

Our study revealed that the new HIV-diagnosis young and middle-aged MSM whose self-rating health was fair/good were less likely to experience persistent moderate–severe depression. They are more confident in their physical state and undergo follow-up antiviral therapy, hence decreasing the likelihood of HIV-related diseases and HIV-related symptoms in life (43), resulting in a low likelihood of unpleasant feelings.

Our study found that self-disclosure of HIV infection predicted the non-depression trajectory. The young and middle-aged MSM who disclosed their HIV status were more likely to be in the non-depression group. Studies have demonstrated that disclosing one’s HIV status to others is a protective factor against depressive symptoms in patients (44, 45). The sexual culture of MSM is relatively open and diversified, and HIV-positive MSM are more likely to disclose their infection status to close friends, regular partners, and family members to obtain more emotional support (46, 47).

We also found that the probability of sustained high-level depression in young and middle-aged MSM with HIV-related symptoms is 3.078 times higher than that in those without HIV-related symptoms. The patients with HIV-related symptoms experienced the discomfort caused by HIV infection and fear of further deterioration of the disease, so they had a relatively high incidence of depressive symptoms. Notably, clinical indicators such as CD_4_^+^ count had no effect on patients in different depression categories in this study. This might be related to the MSM’s stable decrease in CD_4_^+^ count after treatment, resulting in a lower perception of this indicator by the MSM.

There are several limitations in the study. Firstly, the samples in the study were from Beijing, one of the first-tier cities in China. Most of the participants had higher education and higher monthly income, thus, the representativeness of the sample is limited. Secondly, since all data were self-reported, social desirability bias may have contributed to underreporting. Thirdly, similar worded items at five time-points may have influenced the young and middle-aged MSM to reply consistently regardless of real experience. However, by collecting data five times, we can capture a spectrum of depression status of young and middle-aged MSM after HIV-diagnosis to aid the design of target interventions. Finally, this study considered the influence of MSMs’ demographic and clinical characteristics on the trajectory of depressive change, but did not include social factors such as social support and HIV-related stigma. Next, we will combine social factors to comprehensively explore the factors that affect the trajectory of depression changes.

## 5. Conclusion

This study fully considered the group heterogeneity of depression in young and middle-aged MSM with new HIV-diagnosis, and identified three different change tracks: the non-depression group, the mild-depression group and the persistent moderate–severe depression group. The monthly income, self-rating health status, whether to tell others about HIV-infection, and whether having HIV-related symptoms affected depression track categories of the patients. The findings are helpful for medical staff understand the trend of depression in young and middle-aged MSM with different characteristics within one-year after HIV-diagnosis, and to identify individuals with a moderate–severe depression to take target interventions.

## Data availability statement

The raw data supporting the conclusions of this article will be made available by the authors, without undue reservation.

## Ethics statement

The studies involving humans were approved by Beijing Ditan Hospital Capital Medical University. The studies were conducted in accordance with the local legislation and institutional requirements. Written informed consent for participation in this study was provided by the participants’ legal guardians/next of kin.

## Author contributions

XL, YL, and JH designed the work. XL, YL, JH, XB, and FL collected the data. XL and YL analyzed and interpreted the data. XL drafted the manuscript. YL, KL, and JH revised the manuscript. All authors contributed to the article and approved the submitted version.

## Abbreviations

MSM: Men who have Sex with Men
SDS: Self-rating Depression Scale
LCGM: Latent Class Growth Model
GMM: Growth Mixture Models
AIC: Akaike Information Criterion
BIC: Bayesian Information Criterion
aBIC: size-adjusted BIC
LMR: Lo–Mendell–Rubin likelihood ratio test
BLRT: Bootstrapped likelihood ratio test
